# Doxapram Exposure Was Not Associated With Adverse Neurodevelopmental Outcomes in Very Low Birth Weight Infants: A Monocentric Retrospective Cohort Study

**DOI:** 10.1111/apa.70598

**Published:** 2026-05-18

**Authors:** Thomas Müller, Alexandros Rahn, Sabine Pirr, Corinna Peter, Carolin Böhne, Bettina Bohnhorst

**Affiliations:** ^1^ Department of Pediatric Pulmonology, Allergology and Neonatology Hannover Medical School Hannover Germany

**Keywords:** apnea of prematurity, dose dependency, Doxapram, neurodevelopmental outcome

## Abstract

**Aim:**

Doxapram is used as an additional therapy for apnea of prematurity when standard treatments such as caffeine or continuous positive airway pressure are insufficient, but its impact on long‐term neurodevelopment remains uncertain. This study evaluated the association between Doxapram exposure and neurodevelopmental outcomes in very low birth weight infants (VLBWI), with a focus on potential dose‐dependent effects.

**Methods:**

In this monocentric retrospective cohort study, VLBWI with gestational age ≤ 32 weeks were included. Infants treated with Doxapram (*n* = 140) were compared to untreated controls (*n* = 280) matched by year of birth. Exposure was stratified by cumulative dose. The primary outcome was neurodevelopment at 24 months corrected age assessed by the mental development index or global development quotient (MDI/GDQ). Secondary outcomes included mortality, growth parameters and neurological outcomes. Multivariable logistic regression adjusted for confounders.

**Results:**

Neurodevelopment did not differ between groups (MDI/GDQ: 94.2 ± 21.2 vs. 97.7 ± 14.8; *p* = 0.1), nor did mortality (5.0% vs. 6.8%; *p* = 0.53). No dose‐dependent associations were observed. Secondary outcomes were comparable.

**Conclusions:**

Doxapram exposure was not associated with adverse neurodevelopmental outcomes in VLBWI within the investigated dosage range, although definitive conclusions on drug safety cannot be drawn from medium‐sized cohorts and confirmation in prospective randomised studies is required.

## Summary

- Doxapram treats apnea of prematurity when standard therapies are insufficient, but its long‐term neurodevelopmental effects and dose‐dependent toxicity remain poorly understood.
- In this retrospective cohort study of 420 very low birth weight infants, Doxapram exposure was not associated with adverse neurodevelopmental outcomes at 24 months corrected age, regardless of cumulative dose.
- These findings are reassuring, but prospective randomised trials are needed to confirm Doxapram safety.

**Abbreviations**
AOP: apnea of prematurity
BPD: bronchopulmonary dysplasia
BSI: blood stream infection
BSID: Bayley Scales of Infant Development
BW: birth weight
CDD: cumulative Doxapram dose
ConG: control group
CP: cerebral palsy
CPAP: continuous positive airway pressure
DoxG: Doxapram group
ELFRA‐2: Elternfragebögen für die Früherkennung von Risikokindern
FIP: focal intestinal perforation
GA: gestational age
GDQ: global development quotient
GMFCS: gross motor function classification system
HC: head circumference
IQR: interquartile range
IV: intravenous
IVH: intraventricular haemorrhage
MDI: mental development index
NEC: necrotising enterocolitis
NICU: neonatal intensive care unit
PDA: patent ductus arteriosus
PMA: postmenstrual age
ROP: retinopathy of prematurity
SD: standard deviation
VLBWI: very low birth weight infants

## 1 Introduction

Doxapram is a respiratory stimulant used in neonatology for the treatment of apnea of prematurity (AOP), particularly when standard therapies such as caffeine and continuous positive airway pressure (CPAP) are insufficient. Studies from over 20 years ago suggested that Doxapram can negatively affect the psychomotor development of preterm infants and may increase their risk of developmental delays, cerebral palsy (CP) and cognitive impairment [1, 2]. Furthermore, studies have identified short‐term side effects as jitteriness, agitation, arterial hypertension and feeding intolerance [3, 4, 5].

On the contrary, a more recent observational study from the Netherlands did not identify Doxapram as a risk factor for neurological impairment and reported an association to lower mortality among treated preterm infants. However, these studies did not analyse dose dependency, but instead included preterm infants exposed to a wide range of cumulative Doxapram doses (1–1277 mg/kg) [6].

Although Doxapram has demonstrated clear benefits in the treatment of AOP in several studies, its use remains controversial due to a lack of randomised, prospective studies and concerns about adverse effects [3] and it is currently used off‐label in preterm infants.

This study aims to evaluate the association between Doxapram exposure and long‐term neurodevelopmental outcomes, with particular focus on dose‐dependent effects.

We hypothesised that the negative effects of Doxapram on neurodevelopmental outcomes might be dose‐dependent, occurring only above a certain cumulative dose.

## 2 Methods

We conducted a monocentric, retrospective, cohort study at the Neonatal Intensive Care Unit (NICU) of Hannover Medical School. Patient inclusion and collection of epidemiological, clinical and laboratory data were performed using the patient and administration programmes implemented at our NICU (Neodat 5, Version 5.2205.4 and Medipaed Version 5.2019.8; Paedsoft, Tübingen, Germany).

Included were infants born at the Department of Gynaecology and Obstetrics and treated at the NICU between 2006 and 2017 with a gestational age (GA) ≤ 32 weeks and a birth weight (BW) ≤ 1500 g. Exclusion criteria were severe congenital malformations, death within 48 h after birth, intraventricular haemorrhage (IVH) ≥ III°, posthaemorrhagic hydrocephalus requiring intervention and periventricular leukomalacia ≥ II°. All patients treated with Doxapram in the NICU were identified. We excluded those first receiving Doxapram at a GA ≥ 36 weeks for non‐classical AOP indications (e.g., vaccination, anaesthesia). These infants formed the Doxapram group (DoxG). The control group (ConG) consists of preterm infants not treated with Doxapram. To avoid bias from an uneven distribution of birth years between DoxG and ConG and changes in clinical regimens over the study period, two controls matched by year of birth were assigned randomly to each Doxapram infant [7, 8].

To analyse the impact of Doxapram dosage, cumulative Doxapram dose (CDD) per kilogram body weight was calculated for each child as intravenous (IV) equivalent; oral doses were halved based on an assumed gastrointestinal absorption rate of ~50% [9]. DoxG infants were stratified into three groups by CDD: DoxG1 (≤ 75 mg/kg), DoxG2 (> 75–250 mg/kg) and DoxG3 (> 250 mg/kg IV equivalent). The division into the three different dose groups was carried out after calculating the CDD with the aim of obtaining groups of as equal size as possible and to allow exploration of potential dose‐dependent effects. Each DoxG subgroup, as well as the entire DoxG, was compared with ConG. All patients received caffeine citrate as standard of care, at 10 mg/kg/day or 20 mg/kg/day if AOP was insufficiently controlled prior to Doxapram administration.

The primary outcome was neurodevelopment performance at 24 months corrected age. Neurodevelopmental assessment was based on the mental development index (MDI), determined using Bayley Scales of Infant Development (BSID) version II and III. If language barriers, lack of cooperation or disabilities prevented BSID testing, the Griffiths Mental Development Scales, edition II (Global Development Quotient; GDQ), were used. A threshold of MDI or GDQ < 85 defines mild to moderate developmental delay (> 1 standard deviation (SD) below the mean), whereas severe neurodevelopmental impairment was defined as MDI or GDQ < 70 (> 2 SD below the mean) [10, 11, 12]. Secondary outcomes included mortality, body weight, head circumference (HC) at discharge and follow‐up, incidence of CP (GMFCS I or higher, Palisano definition), visual impairment (need for glasses), hearing impairment (need for hearing aids) and speech developmental delay at follow‐up, defined as an abnormal result on the ELFRA‐2 (‘Elternfragebögen für die Früherkennung von Risikokindern’). The primary comparison was performed between DoxG and the untreated ConG. Analyses of dose‐dependent effects included comparisons between ConG and each of the three Doxapram dose subgroups as well as comparisons among these dose subgroups. Additional analyses comparing infants with severe neurodevelopmental impairment (MDI/GDQ < 70) with those without severe impairment (MDI/GDQ ≥ 70) were performed.

Internal follow‐up, including neurodevelopmental testing, was performed by a trained neonatologist at Hannover Medical School. External follow‐up results came from the Lower Saxony follow‐up project, which centrally collected the data. The formal approval of the responsible commission had been granted.

Ethical approval was obtained from the ethics commission and data protection officer of Hannover Medical School (No. 10126_BO_K_2021). It was designed to use anonymous patient data so that no one could be identified.

### 2.1 Statistical Analysis

Demographic, perinatal and clinical parameters were processed in Excel (version 2018, Microsoft Corporation, Washington, DC, USA). Statistical analyses were performed with GraphPad Prism (v9.4.0 (453), San Diego, California, USA). The Shapiro–Wilk test was used to test for normality. Normally distributed data were presented as mean ± SD, while non‐normally distributed data were given as median and interquartile range (IQR; 25–75th percentile). Categorical values were analysed by Fisher's exact test; continuous variables by unpaired *t*‐test or Mann–Whitney *U* test as appropriate. ANOVA with Tukey's post hoc test and Kruskal‐Wallis test followed by Dunn's post hoc test with Bonferroni correction were used for group comparisons. To adjust for confounders influencing neurodevelopment, multiple logistic regression analysis was performed, including the following as independent confounders: GA, BW, IVH ≤ II°, insulin‐dependent hyperglycemia, sedation > 48 h, duration of invasive ventilation, moderate/severe bronchopulmonary dysplasia (BPD), blood culture proven sepsis, necrotising enterocolitis ≥ IIa (NEC) and focal intestinal perforation (FIP), microcephaly (< 3rd percentile) and hypotrophy (< 10th percentile) at discharge and Doxapram treatment. *p* < 0.05 was deemed significant.

## 3 Results

### 3.1 Study Population and Baseline Characteristics

**FIGURE 1:**
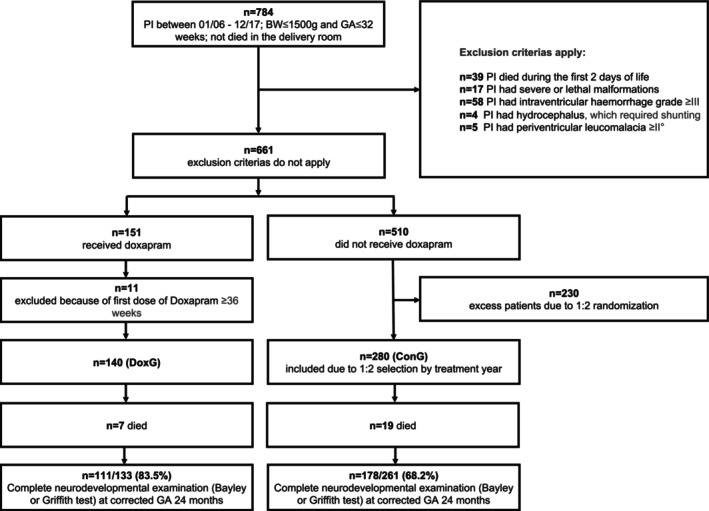
Cohort flow chart: Inclusion, exclusion and Doxapram group assignment. All preterm infants (PI) ≤ 32 weeks of gestational age (GA) and ≤ 1500 g birth weight (BW) who did not die in the delivery room were screened for eligibility. After applying predefined exclusion criteria, eligible PI were assigned to the Doxapram (DoxG) or the control group (ConG). The PI of the ConG were selected due to a 2:1 random sampling matched by year of birth.

Figure 1 shows the recruitment process and epidemiological, clinical and laboratory characteristics are shown in Table 1. The final cohort included 140 very low birth weight infants (VLBWI, GA ≤ 32 weeks) in DoxG and 280 VLBWI in ConG. Of these, 7 infants in DoxG and 19 infants in ConG died during hospital stay (DoxG: 7/140 (5.0%), ConG: 19/280 (6.8%), *p* = 0.53). Complete neurodevelopmental follow‐up at 24 months corrected age were available for 111/133 DoxG infants (83.5%) and 178/261 ConG infants (68.2%). DoxG infants had significantly lower median GA (25.7 vs. 29.4 weeks), lower median BW (0.77 vs. 1.13 kg) and smaller median HC (23 vs. 26.8 cm) compared to ConG (all *p* < 0.0001). They also had significant higher rates of respiratory complications, greater need for catecholamines and sedation, increased rates of patent ductus arteriosus (PDA) requiring treatment, FIP, IVH ≤ II°, blood stream infection (BSI), more surgeries, transfusion‐requiring anaemia and retinopathy of prematurity (ROP) requiring intervention. Median hospital stay was significantly longer in DoxG (96 vs. 53 days, *p* < 0.0001). Additional clinical data are provided in Table S1.

**TABLE 1:** Demographic and clinical characteristics of the study cohort: Doxapram versus control group.

Parameter	Doxapram group (*n* = 140)	Control group (*n* = 280)	*p*
Gestational age [weeks]	25.7 (24.6–27.1)	29.4 (27.7–30.6)	**< 0.0001** ^a^
Birth weight [kg]	0.77 (0.62–0.95)	1.13 (0.92–1.33)	**< 0.0001** ^a^
Head circumference at birth [cm]	23 (21.7–25)	26.8 (25–28)	**< 0.0001** ^a^
Female (%)	52 (37.1)	135 (48.2)	**0.04** ^b^
RDS grade	2 (2–3), *n* = 139	2 (1–2), *n* = 266	**< 0.0001** ^a^
Surfactant treatment (%)	102 (72.9)	100 (35.7)	**< 0.0001** ^b^
Ventilation duration invasive + non‐invasive mechanical ventilation: S‐IMV + HFO + CPAP [h]	1318 (874–1629)	154 (52–457)	**< 0.0001** ^a^
Ventilation duration invasive mechanical ventilation: S‐IMV + HFO [h]	144 (23–365)	0 (0–43)	**< 0.0001** ^a^
Ventilation duration non‐invasive mechanical ventilation: CPAP [h]	1062 (711–1342)	117 (30–417)	**< 0.0001** ^a^
Sedation required (Morphin and/or Fentanyl and/or Midazolam) > 48 h (%)	22 (15.7)	20 (7.1)	**0.009** ^b^
Dexamethasone treatment (%)	41 (29.3)	12 (4.3)	**< 0.0001** ^b^
Moderate or severe BPD (%)	60 (45.5), *n* = 132	22 (8.4), *n* = 261	**< 0.0001** ^b^
Catecholamine treatment (%)	52 (37.1)	41 (14.6)	**< 0.0001** ^b^
PDA requiring treatment (%)	75 (53.6)	49 (17.5)	**< 0.0001** ^b^
IVH ≤ II° or cerebellar haemorrhage (%)	39 (27.9)	22 (7.9)	**< 0.0001** ^b^
Blood stream infection (%)	26 (18.6)	26 (9.3)	**0.** **01** ^b^
Clinical sepsis (%)	74 (52.9)	41 (14.6)	**< 0.** **0001** ^b^
Number of FIP (%)	7 (5.0)	2 (0.7)	**0.008** ^b^
Number of operations in general anaesthesia (%)	48 (34.3)	18 (6.4)	**< 0.** **0001** ^b^
Number of erythrocyte concentrates because of anaemia	3 (1–5)	0 (0–1)	**< 0.0001** ^a^
ROP requiring treatment (%)	9 (6.8), *n* = 133	1 (0.4), *n* = 236	**0.0006** ^b^
Length of hospital stay [days]	96 (81–110), *n* = 133	53 (40–68), *n* = 261	**< 0.0001** ^a^
Mortality (%)	7 (5.0)	19 (6.8)	0.53^b^

*Note:* Total cohort: *n* = 420; missing data are indicated where applicable. Due to the non‐normal distribution, data are presented as median and interquartile range. A *p*‐value < 0.05 was considered statistically significant and is shown in bold.

Abbreviations: BPD = bronchopulmonary dysplasia as defined by Walsh et al., *J Perinatol*. 2003, CPAP = continuous positive airway pressure, FIP = focal intestinal perforation, HFO = high frequency oscillation, IVH = intraventricular haemorrhage, PDA = patent ductus arteriosus, RDS = respiratory distress syndrome, ROP = retinopathy of prematurity, S‐IMV = synchronised intermittent mandatory ventilation.

^a^ Mann–Whitney *U*‐test.

^b^ Fisher's exact test.

### 3.2 Doxapram Application

The three Doxapram groups (DoxG1, DoxG2, DoxG3) were approximately equal in size. Postnatal age at first administration did not differ significantly between groups, but cumulative dose and duration of treatment did; DoxG1 had a shorter duration than DoxG2 and DoxG3 and DoxG2 had a shorter duration than DoxG3 (*p* < 0.0001). Doxapram was initiated at postmenstrual ages from 24.1 to 33.6 weeks (median 28 weeks). DoxG3 infants received Doxapram at a lower GA than DoxG1 and DoxG2 (27 vs. 29 and 28 weeks, *p* < 0.025). Importantly, the mode of Doxapram therapy (dose, GA/initiation, age at first use, duration) showed no significant effect on the developmental outcome. Doxapram treatment modalities of the individual Doxapram dose subgroups are provided in Table 2.

**TABLE 2:** Comparison of Doxapram treatment modalities by dosing subgroups.

Application modality	Doxapram group (*n* = 140)	DoxG1 (*n* = 53)	DoxG2 (*n* = 42)	DoxG3 (*n* = 45)	*p*
Chronological age at first administration [days of life]	13.5 (1–45)	13 (1–37)	18.5 (1–45)	12 (1–31)	ns
Cumulative dose [mg/kg IV equivalent]	122 (3–1589)	35 (3–74)	134.1 (79–250)	420 (253–1589)	**All** < **0.0001**
Days of application	10 (1–57)	3 (1–11)	11.5 (3–26)	23 (9–57)	**All < 0.0001**
PMA at first administration [weeks]	28 (24–34)	29 (25–33)	28 (25–34)	27 (24–31)	*

*Note:* Patients were stratified into three groups based on cumulative intravenous (IV) equivalent dose: DoxG1 (≤ 75 mg/kg), DoxG2 (> 75–250 mg/kg), DoxG3 (> 250 mg/kg). Values are presented as median (range: minimum—maximum) due to non‐normal distribution. Statistical comparisons were performed using the Kruskal‐Wallis test, followed by Dunn's post hoc test with Bonferroni correction for multiple pairwise comparisons between the three subgroups. Adjusted *p*‐values are reported. A *p‐va*< 0.05 was considered statistically significant and is shown in bold.

Abbreviation: PMA = postmenstrual age.

* DoxG1 versus DoxG2: *p* = 0.23; DoxG1 versus DoxG3: *p* < 0.0001; DoxG2 versus DoxG3: *p* = 0.025.

### 3.3 Primary Outcome

**FIGURE 2:**
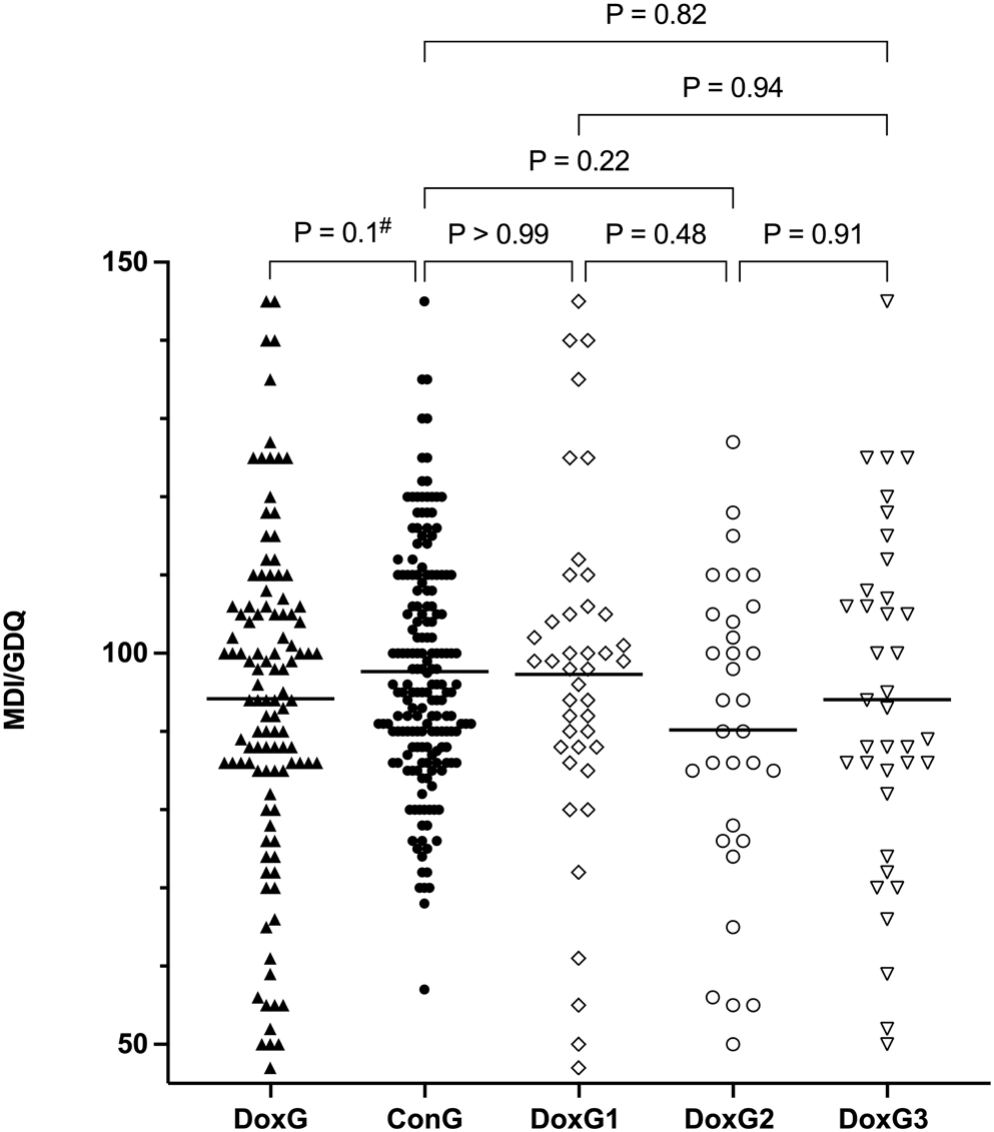
Scatter dot plot of the composite score of the Mental Development Index (MDI; Bayley‐II and III) and the Griffith's Developmental Quotient (GDQ; Griffith's Scales of Child Development) across the five groups. Control group (ConG), overall Doxapram group (DoxG) and three Doxapram dose subgroups—DoxG1 (≤ 75 mg/kg), DoxG2 (> 75–250 mg/kg) and DoxG3 (> 250 mg/kg). Each dot represents one patient; horizontal lines indicate group means. One‐way ANOVA showed no statistically significant differences between ConG and DoxG subgroups (all *p* > 0.05). Group sizes: ConG (*n* = 178), DoxG (*n* = 111), DoxG1 (*n* = 42), DoxG2 (*n* = 32), DoxG3 (*n* = 37). # = independent samples *t*‐test was used.

The primary outcome was neurodevelopmental performance at 24 months corrected age. Mean MDI or GDQ values were within the normal range (85–115) in both groups and did not differ significantly between the DoxG and the ConG (94.2 ± 21.2 vs. 97.7 ± 14.8; *p* = 0.1; see Figure 2). Similarly, no significant differences were observed between ConG and the individual Doxapram dose subgroups (see Table 3). As DoxG2 showed a trend towards lower neurodevelopmental scores, additional analyses were performed to further characterise this subgroup. Differences in baseline characteristics between DoxG2 and the remaining cohort are presented in Table S2. Distribution of developmental test types was comparable between groups (see Table S3).

**TABLE 3:** Cognitive, motor and sensory outcomes in Control and Doxapram groups after 24 months corrected age, including dose subgroup analysis.

Parameter	Doxapram group (*n* = 140)	Control group (*n* = 280)	DoxG1 (*n* = 53)	DoxG2 (*n* = 42)	DoxG3 (*n* = 45)	*p* (DoxG vs. ConG)	*p* (ConG vs. DoxG 1–3)
MDI/GDQ	94.2 ± 21.2, *n* = 111	97.7 ± 14.8, *n* = 178	97.3 ± 21.8, *n* = 42	90.2 ± 19.6, *n* = 32	94.1 ± 21.6, *n* = 37	0.1	ns
Cerebral palsy (%)	4/118 (3.4)	2/189 (1.1)	2/46 (4.3)	2/34 (5.9)	0/48 (0)	0.21	ns
Visual impairment (%)	10/121 (8.3)	7/189 (3.7)	1/47 (2.1)	5/36 (13.9)	4/38 (10.5)	0.12	ns
Hearing impairment (%)	1/121 (0.8)	3/189 (1.6)	1/47 (2.1)	0/36 (0)	0/38 (0)	1	ns
Language developmental delay (%)	50/104 (48.1)	78/177 (44.1)	17/38 (44.7)	14/31 (45.2)	19/35 (54.3)	0.54	ns

*Note:* Neurodevelopmental outcomes are presented for the Control group (ConG), the overall Doxapram group (DoxG) and for the Doxapram subgroups stratified by cumulative intravenous equivalent dose: DoxG1 (≤ 75 mg/kg), DoxG2 (> 75–250 mg/kg), DoxG3 (> 250 mg/kg). Values are presented as mean ± SD due to normal distribution. Missing data are indicated where applicable. The composite score of the Mental Development Index (MDI) from the Bayley Scales of Infant Development II and III, and the Griffith's Developmental Quotient (GDQ) from the Griffiths Mental Development Scales was normally distributed and analysed using an independent samples *t*‐test for comparisons between ConG and DoxG, and one‐way ANOVA for comparisons between ConG and the three dose subgroups, followed by Tukey's post hoc test. All other outcomes were categorical and were analysed using Fisher's exact test for comparisons between ConG and DoxG, and Fisher's exact test for multi‐group comparisons (ConG vs. DoxG1‐3). Posthoc pairwise testing with Bonferroni correction was applied. Adjusted *p*‐values are reported. A *p*‐value < 0.05 was considered statistically significant. Cerebral palsy was defined as a score ≥ 1 according to the Gross Motor Function Classification System (GMFCS). Visual impairment was defined as the need for corrective glasses; hearing impairment as the requirement for a hearing device. Language developmental delay was defined as an abnormal result on the ‘Elternfragebögen für die Früherkennung von Risikokindern’ (ELFRA‐2), a standardised parent‐report screening questionnaire for early language development.

### 3.4 Secondary Outcomes

At discharge, both median body weight and HC were significantly higher in DoxG (2.81 kg [2.51–3.24] and 33.5 cm [32.5–34.5], respectively) than in ConG (2.39 kg [2.12–2.81] and 32.5 cm [31.5–34.0]; *p* < 0.0001). At follow up (24 months corrected age), body weight did not differ between groups (DoxG: 11.0 kg [10.1–12.2]; ConG: 11.4 kg [10.5–12.5], *p* = 0.16), whereas HC was slightly smaller in DoxG (47.5 ± 1.57 cm vs. 47.9 ± 1.66 cm, *p* = 0.047). Rates of CP, visual/hearing impairment and speech developmental delays did not differ significantly between groups. No cases of deafness or blindness were reported in either group. Neurodevelopmental data are shown in Table 3, and exploratory data on psychomotor development are presented in Table S4.

### 3.5 Multivariable Analysis

In multiple logistic regression analysis, neurodevelopmental impairment (MDI/GDQ < 85; > 1 SD below the mean) was used as the dependent variable. NEC (> IIa)/FIP, prolonged invasive ventilation and hypotrophy (body weight < 10th percentile) at discharge were identified as independent risk factors for adverse neurodevelopmental outcome (see Table 4), whereas Doxapram therapy itself showed no significant association.

**TABLE 4:** Multiple logistic regression of risk for neurodevelopmental delay defined as Mental Development Index or Griffith's Developmental Quotient > 1 standard deviation below the normative mean (< 85).

Parameter	Odds ratio	95%‐confidence interval	*p*
Moderate or severe BPD	1.0	0.4–2.5	0.9
Doxapram	1.1	0.5–2.4	0.9
Blood culture proven sepsis	0.8	0.2–4.1	0.7
IVH ≤ II°	1.2	0.6–2.1	0.6
Hyperglycemia requiring insulin	0.7	0.2–1.9	0.5
Sedation > 48 h	0.5	0.1–1.6	0.3
Birth weight (kg)	6.6	0.6–75.1	0.1
Gestational age (weeks)	0.8	0.6–1.1	0.1
Head circumference at discharge ≤ 3rd percentile (%)	2.1	1.0–4.4	0.06
NEC (> IIa)/FIP	4.5	1.004–21.5	**0.049**
Prolonged invasive ventilation (h)	1.002	1.0–1.004	**0.03**
Weight at discharge < 10th percentile (%)	2.9	1.3–6.3	**0.008**

*Note:* Results of the multiple logistic regression analysis of different clinical parameters as potential confounders for abnormal neurological outcome in preterm infants. A *p*‐value < 0.05 was considered statistically significant and is shown in bold.

Abbreviations: BPD = bronchopulmonary dysplasia as defined by Walsh et al., *J Perinatol*. 2003, FIP = focal intestinal perforation, IVH = intraventricular haemorrhage, NEC = necrotising enterocolitis.

### 3.6 Analysis of Infants With Severe Neurodevelopmental Impairment

As illustrated in Figure 2, the DoxG showed a greater spread of neurodevelopmental scores with a higher number of low outliers, resulting in a higher variance (SD 21.2 vs. 14.8 in ConG), consistent with the greater clinical heterogeneity of this group. To investigate which factors may explain the low scores in this group, additional analyses were performed comparing infants with severe neurodevelopmental impairment (MDI/GDQ < 70; > 2 SD below the mean) with infants with MDI/GDQ ≥ 70. Within the doxapram‐treated group, infants with MDI/GDQ < 70 differed from those with MDI/GDQ ≥ 70 with respect to several neonatal characteristics, including BW, duration of invasive ventilation, rates of IVH ≤ II°, BSI, NEC (> IIa)/FIP and body weight below the 10th percentile at discharge (see Table S5). The same comparison was performed in the entire study cohort, irrespective of doxapram exposure, revealing a similar pattern (Table S6). These findings suggest that the greater spread of scores in the doxapram group is likely attributable to the higher burden of neonatal morbidity in this group, rather than to doxapram exposure itself, which was not identified as an independent risk factor in the multivariable analysis (Table 4).

## 4 Discussion

To date, this is one of the largest studies to examine the effect of Doxapram on long‐term neurodevelopmental outcomes in VLBWI with GA ≤ 32 weeks and to our knowledge, the first to assess these outcomes in relation to different cumulative Doxapram dosages.

In this retrospective cohort, no evidence was found for an adverse effect of Doxapram on neurodevelopmental outcomes at 24 months corrected age, for cumulative doses up to 1589 mg/kg IV equivalent. Infants in DoxG represented a more vulnerable population with lower GA, lower BW and higher morbidity; these differences should be considered when interpreting the results. Despite this higher baseline risk, neurodevelopmental outcomes were comparable between groups, although residual confounding cannot be excluded. While head circumference was statistically smaller in DoxG (mean difference −0.4 cm), the clinical relevance of this finding appears limited. Furthermore, no association was observed between cumulative Doxapram dose or treatment characteristics and neurodevelopmental outcome.

Doxapram, first synthesised in 1962, is a dose‐dependent respiratory stimulant enhancing both respiratory rate and tidal volume, therefore reducing pCO_2_. Its effect mechanism involves stimulation of brainstem respiratory centres as well as peripheral carotid and aortic chemoreceptors, and it may improve respiratory muscle function [13]. Initially used in anesthesiology due to a greater safety margin, Doxapram has since the early 1980s established itself in the management of AOP [14, 15, 16]. Although Doxapram's efficiency for treatment of AOP—alone or in combination with CPAP and/or caffeine—is widely recognised, persistent concerns regarding safety exist. Notably, the linkage between Doxapram and developmental disorders is largely based on a retrospective case–control study involving 80 preterm infants (BW < 1250 g; 40 with isolated mental developmental delay; 40 with normal mental development matched for GA, BW, IVH grade and socioeconomic status) published in 2001 [1]. Infants with developmental delay received substantially higher Doxapram doses (2233 ± 1927 mg vs. 615 ± 767 mg) and longer exposures (45.2 ± 32.5 vs. 19.4 ± 23.4 days, *p* < 0.001). No multivariable regression analysis adjusting for postnatal morbidity or cumulative disease severity was performed. In addition, infants had been treated with excessively high doses and formulations containing benzyl alcohol, a neurotoxin contraindicated in neonates. It is conceivable that these findings reflected underlying disease severity rather than a direct drug effect. Moreover, clinical practices in the 1990s differ from today. In contrast to this early case–control study, we deliberately did not perform matching for GA or BW; instead, these variables and other markers of neonatal morbidity were accounted for by multivariable logistic regression. In addition, analyses showed that infants with severe neurodevelopmental impairment (MDI/GDQ < 70) had a higher burden of neonatal morbidity, including factors that are well established risk factors for neurodevelopmental delay, while no association between Doxapram exposure and adverse neurodevelopmental outcomes was observed within the investigated dosage range.

Lando et al. provide further data from structured telephone interviews done by the NICU staff with parents of 53 preterm babies (GA < 27 weeks, follow‐up at 7–16 months corrected age). The interview based on the German version of the Revised Pre‐Screening Developmental Questionnaire. Infants showed a mean general developmental score of 14.9 ± 3.9. For each 10 days of Doxapram therapy a 0.26 ± 0.11 points decrease in the developmental score was seen, which the authors judged only marginally relevant, suggesting minimal impact on neurodevelopment [2]. More recent reports have yielded contrary results: in 2016, Ten Hove et al. published a retrospective cohort study comparing 142 Doxapram‐treated infants to 284 non‐treated controls (GA < 30 weeks, BW < 1250 g) and reported a significantly lower risk of death or neurodevelopmental delay in the treated group (OR = 0.54, 95% CI: 0.37–0.78), albeit with increased BPD risk [6]. However, in 2017, the same group conducted a systematic review of four small randomised trials and 28 observational studies, highlighting substantial heterogeneity and ultimately concluding that Doxapram's safety and efficacy remain uncertain—recommending its use only in well‐conducted, randomised, placebo‐controlled trials [3].

The largest and most robust dataset to date derives from the french EPIPAGE‐2 cohort, in which 821 preterm infants were included in a 2:1 propensity score‐matched analysis (median GA 29.4 weeks [IQR 27.6–30.9]). They found no differences in survival or neurodevelopmental disabilities of any severity (OR 1.09, 95% CI: 0.76–1.57, *p* = 0.63), nor for moderate/severe overall neurodevelopmental disabilities (OR 1.08, CI: 0.75–1.55, *p* = 0.67) at ages 5–6 years, regardless of Doxapram exposure [17]. However, dosage details were not reported, treatment duration was shorter (maximum 24 days) and infants had a higher median GA than in our cohort. Our study provides additional data on neurodevelopmental outcomes in Doxapram‐treated preterm infants. Notably, in our cohort DoxG infants had significantly lower GA and BW than controls, reflecting an indication‐related group difference, as less mature infants are at greater risk of AOP and therefore more likely to receive Doxapram. Neurodevelopmental and survival outcomes should be interpreted in this clinical context, as comparable outcomes were observed despite the higher baseline vulnerability of the Doxapram group, which may be considered reassuring. Further research is needed to clarify the effects of Doxapram on long‐term neurodevelopmental outcomes, including randomised controlled trials such as the ongoing DOXA‐trial [18].

## 5 Limitations

Our findings should be interpreted in light of several limitations. First, dosage groups may overlap within the borderline range of dosage intervals due to the assumption of 50% bioavailability for oral administration. True absorption range may be lower or higher (up to 74%), potentially skewing results [9, 19].

Second, DoxG infants were more immature and experienced significantly more short‐term morbidity (e.g., prolonged invasive ventilation, NEC/FIP/BSI, IVH, sedation, dexamethasone therapy), all known risk factors for developmental delay [20, 21, 22]. We accounted for these variables via multiple regression analyses, but residual confounding cannot be excluded due retrospective design of our study, which may have influenced the comparability of the groups. Notably, we did not match GA and BW, as 1:1 matching increases selection bias and reduces the available controls, especially with additional matching variables.

Third, although DoxG2 showed a trend towards lower neurodevelopmental scores, significant differences in baseline characteristics should be considered when interpreting these results.

Fourth, three different developmental tests (Griffith and BSID II/III) were employed, partly due to the twelve‐year timeframe. Literature has addressed possible differences between the tests. Some studies found a stronger correlation between the Griffith and BSID II, while others report a higher level of agreement between Griffith and BSID III. In principle, higher developmental scores are achieved with BSID III but still reliably identify children needing medical intervention [23, 24, 25]. As test type usage was balanced across groups, we consider effects minimal.

Fifth, psychomotor development could not be comprehensively analysed due to limited data availability, and results should therefore be interpreted with caution.

Sixth, the lower follow‐up rate in ConG (68.2% vs. 83.5% in DoxG) represents a potential source of bias. If healthier infants in ConG were less likely to attend follow‐up, the observed neurodevelopmental scores in this group may not fully reflect the true distribution of outcomes, potentially affecting the comparability between groups. However, the reasons for non‐attendance were not available in our dataset and the direction of this potential bias therefore remains uncertain. Future studies should aim for complete follow‐up or systematically document reasons for non‐attendance to allow for sensitivity analyses.

Finally, data on maternal education and social status of the family were unavailable, so their influence could not be evaluated.

## 6 Conclusions

In conclusion, our findings show no evidence of an adverse association between Doxapram exposure within the investigated dosage range and neurodevelopmental outcomes in VLBWI. These results should be interpreted with caution given the observational study design, baseline differences between groups and the inherent limitation that definitive conclusions on drug safety are difficult to establish in medium‐sized cohorts. Further prospective and randomised studies are urgently needed to confirm our findings.

## Author Contributions

**Corinna Peter:** writing – review and editing, validation. **Sabine Pirr:** formal analysis, data curation, writing – review and editing. **Carolin Böhne:** writing – review and editing, investigation. **Bettina Bohnhorst:** conceptualization, investigation, writing – original draft, writing – review and editing, visualization, methodology, validation, formal analysis, project administration, supervision, data curation. **Alexandros Rahn:** conceptualization, investigation, writing – original draft, validation, methodology, visualization, writing – review and editing, formal analysis, data curation, software. **Thomas Müller:** conceptualization, investigation, writing – original draft, methodology, validation, visualization, writing – review and editing, formal analysis, data curation, software.

## Funding

The authors have nothing to report.

## Ethics Statement

The study was approved by the Ethics Committee of Hannover Medical School (No. 10126_BO_K_2021) and conducted in accordance with the ethical principles of the 1964 Declaration of Helsinki. Due to the retrospective design and use of anonymised data, the requirement for written informed consent was waived by the ethics committee.

## Consent

The manuscript does not contain any individual person's data in any form.

## Conflicts of Interest

The authors declare no conflicts of interest.

## Supporting information


**Table S1:** Detailed demographic and clinical characteristics of the study cohort: Doxapram versus control group.
**Table S2:** Comparison of confounders for neurodevelopment outcome between DoxG2 and all other investigated patients.
**Table S3:** Number of neurodevelopment assessment tests applied in each group.
**Table S4:** Psychomotor developmental outcomes in ConG and DoxG groups after 24 months corrected age, including dose subgroup analysis.
**Table S5:** Clinical characteristics of Doxapram‐treated preterm infants with MDI/GDQ ≥ 70 compared with those with severe neurodevelopmental impairment (MDI/GDQ < 70; > 2 SD below the mean).
**Table S6:** Clinical characteristics of the overall study cohort, comparing preterm infants with MDI/GDQ ≥ 70 and those with severe neurodevelopmental impairment (MDI/GDQ < 70; > 2 SD below the mean), irrespective of Doxapram exposure.

## Data Availability

The datasets generated and analysed during this study are not publicly available due to patient confidentiality regulations but are available from the corresponding author upon reasonable request.
