# Supplementary material for: Doxapram Exposure Was Not Associated With Adverse Neurodevelopmental Outcomes in Very Low Birth Weight Infants: A Monocentric Retrospective Cohort Study
Source: Acta Paediatr. 2026 May 18;115(9):1982–90. doi: 10.1111/apa.70598 (PMC13460644; doi:10.1111/apa.70598)
Supplement: Supplementary file 1 [file APA-115-1982-s001.docx]

**TABLE S1**. Detailed demographic and clinical characteristics of the study cohort: Doxapram versus control group

| **Parameter** | | | **Doxapram group (n=140)** | **Control group (n=280)** | **P-value** |  |  |
| --- | --- | --- | --- | --- | --- | --- | --- |
| Gestational age [weeks] | | | 25.7 (24.6 - 27.1) | 29.4 (27.7 - 30.6) | **<0.0001^†^** |  |  |
| Birth weight [kg] | | | 0.77 (0.62 - 0.95) | 1.13 (0.92 - 1.33) | **<0.0001^†^** |  |  |
| Small for gestational age [<10^th^ percentile] (%) | | | 21 (15.0) | 63 (22.5) | 0.35^‡^ |  |  |
| Weight at discharge [kg] | | | 2.81 (2.51 - 3.24), n=133 | 2.39 (2.12 - 2.81), n=261 | **<0.0001^†^** |  |  |
| Weight at discharge <10^th^ percentile (%) | | | 62 (46.6), n=133 | 117 (44.8), n=261 | 0.75^‡^ |  |  |
| Follow-up weight at 24 month corrected age [kg] | | | 11.0 (10.1 – 12.2), n=117 | 11.4 (10.5 – 12.5), n=193 | 0.16^†^ |  |  |
| Follow-up weight at 24 month corrected age <10^th^ percentile (%) | | | 35 (30.7) n=117 | 44 (15.7), n=193 | 0.18^‡^ |  |  |
| Head circumference at birth [cm] | | | 23 (21.7 - 25) | 26.8 (25 - 28) | **<0.0001^†^** |  |  |
| Head circumference at birth ≤ 3^rd^ percentile | | | 9 (6.4) | 16 (5.7) | 0.83^‡^ |  |  |
| Head circumference at discharge [cm] | | | 33.5 (32.5 - 34.5), n=133 | 32.5 (31.5 - 34.0), n=261 | **<0.0001^†^** |  |  |
| Head circumference at discharge ≤ 3^rd^ percentile | | | 47 (35.3), n=133 | 53 (20.3), n=261 | **0.0015^‡^** |  |  |
| Follow-up head circumference at 24 month corrected age [cm] | | | 47.5 ± 1.57, n=117 | 47.9±1.66, n=193 | **0.047*** |  |  |
| Follow-up head circumference at 24 month corrected age ≤ 3^rd^ percentile | | | 37 (33.0), n=117 | 45 (24.6), n=192 | 0.14^‡^ |  |  |
| Female (%) | | | 52 (37.1) | 135 (48.2) | **0.04^‡^** |  |  |
| Caucasian (%) | | | 87 (84.5), n=103 | 118 (80.8), n=146 | 0.5^‡^ |  |  |
| 5min APGAR | | | 7.5 (7 - 8) | 8 (7 - 9) | **<0.0001^†^** |  |  |
| 10min APGAR | | | 8 (8 - 9) | 9 (8 - 9) | **<0.0001^†^** |  |  |
| pH umbilical artery | | | 7.34 (7.28 - 7.38) | 7.31 (7.26 - 7.36) | 0.051^†^ |  |  |
| Base excess umbilical artery [mmol/l] | | | -2.9 (-5.6 to -1.1) | -3.4 (-6.2 to -1.4) | 0.26^†^ |  |  |
| Temperature at admission [°C] | | | 36.9 (36.5 - 37.2) | 36.8 (36.6 - 37.1) | 0.99^†^ |  |  |
| RDS grade | | | 2 (2 - 3), n=139 | 2 (1 - 2), n=266 | **<0.0001^†^** |  |  |
| Surfactant treatment (%) | | | 102 (72.9) | 100 (35.7) | **<0.0001^‡^** |  |  |
| Ventilation duration invasive + non-invasive mechanical ventilation: S-IMV+HFO+CPAP [h] | | | 1318 (874 - 1629) | 154 (52 - 457) | **<0.0001^†^** |  |  |
|  |  |  |  |  |  |  |  |
|  | | |  |  |  |  |  |

| Ventilation duration invasive mechanical ventilation: S-IMV+HFO [h] | 144 (23 - 365) | 0 (0 - 43) | **<0.0001^†^** |
| --- | --- | --- | --- |
| Ventilation duration non-invasive mechanical ventilation: CPAP [h] | 1062 (711 - 1342) | 117 (30 - 417) | **<0.0001^†^** |
| Dexamethasone treatment (%) | 41 (29.3) | 12 (4.3) | **<0.0001^‡^** |
| Moderate or severe BPD (%) | 60 (45.5), n=132 | 22 (8.4), n=261 | **<0.0001^‡^** |
| NO treatment (%) | 14 (10.0) | 16 (5.7) | <0.11^‡^ |
| Catecholamine treatment (%) | 52 (37.1) | 41 (14.6) | **<0.0001^‡^** |
| Hyperglycemia requiring insulin (%) | 34 (24.3) | 17 (6.1) | **<0.0001^‡^** |
| PDA requiring treatment (%) | 75 (53.6) | 49 (17.5) | **<0.0001^‡^** |
| IVH ≤II° or cerebellar hemorrhage (%) | 39 (27.9) | 22 (7.9) | **<0.0001^‡^** |
| Blood stream infection (%) | 26 (18.6) | 26 (9.3) | **0.01^†^** |
| Clinical sepsis (%) | 74 (52.9) | 41 (14.6) | **<0.0001^†^** |
| CoNS sepsis^§^ | 8 (5.7) | 3 (1.1) | **0.008 ^†^** |
| Number of operations in general anesthesia (%) | 48 (34.3) | 18 (6.4) | **<0.0001^†^** |
| Number of erythrocyte concentrates because of anemia | 3 (1 - 5) | 0 (0 - 1) | **<0.0001^†^** |
| ROP > 2° | 13 (9.8), n=133 | 1 (0.4) n=236 | **<0.0001^‡^** |
| ROP requiring treatment (%) | 9 (6.8), n=133 | 1 (0.4), n=236 | **0.0006^‡^** |
| Length of hospital stay [days] | 96 (81 - 110), n=133 | 53 (40 - 68), n=261 | **<0.0001^†^** |
| Post menstrual age at discharge [weeks] | 39.1 (38.3 - 40.9) | 37.0 (36.0 - 38.0) | **<0.0001^†^** |
| Mortality (%) | 7 (5.0) | 19 (6.8) | 0.53^‡^ |
| NEC > IIa (%) | 8 (5.7) | 7 (2.5) | 0.1^‡^ |
| FIP (%) | 7 (5.0) | 2 (0.7) | **0.008^‡^** |
| Sedation required (Morphin. Fentanyl and/or Midazolam) > 48h (%) | 22 (15.7) | 20 (7.1) | **0.009^‡^** |
| Abnormal BERA at discharge (%) | 16 (13.0), n=123 | 31 (13.1), n=236 | >0.99^‡^ |

Total cohort: n = 420; missing data are indicated where applicable. Normally distributed values are presented as mean ± standard deviation, non-normally distributed as median and interquartile range. Statistical tests used: *Unpaired t-Test, ^†^Mann-Whitney U-test, ^‡^Fisher’s exact test. p < 0.05 was considered statistically significant and is shown in bold. BERA = brainstem electric response audiometry; BPD = bronchopulmonary dysplasia as defined by Walsh MC, Wilson-Costello D, Zadell A, et al., J Perinatol. 2003; CoNS = coagulase-negative staphylococci; CPAP = continuous positive airway pressure; FIP = focal intestinal perforation; HFO = high frequency oscillation; IVH = intraventricular hemorrhage; NEC = necrotizing enterocolitis; NO = nitrogen monoxide; PDA = patent ductus arteriosus; RDS = respiratory distress syndrome; ROP = retinopathy of prematurity; S-IMV = synchronized intermittent mandatory ventilation.

**TABLE S2.** Comparison of confounders for neurodevelopment outcome between DoxG2 and all other investigated patients

| **Clinical Parameter** | **DoxG2 (n=42)** | **ConG+DoxG1+3 (n=378)** | **P-value** |
| --- | --- | --- | --- |
| Gestational age [weeks] | 25.7 (24.7-26.6) | 28.6 (26.4-30.1) | **<0.0001^†^** |
| Moderate or severe BPD (%) | 21 (52.5), n=40 | 61 (17.2), n=354 | **<0.0001^‡^** |
| Duration of invasive ventilation [h] ^§^ | 234 ± 239 (0-972) | 103±274 (0-3372) | **<0.0001^†^** |
| Blood stream infection^§^ | 14 (33.3) | 49 (13.0) | **0.0014^†^** |
| IVH ≤II° or cerebellar hemorrhage (%) | 11 (26.2) | 45 (11.9) | **0.0158^‡^** |
| Head circumference at discharge ≤ 3^rd^ percentile (%) | 17 (42.5), n=40 | 91 (25.7), n=354 | **0.038^†^** |
| Weight at discharge <10^th^ percentile (%) | 24 (60.0), n=40 | 153 (43.2), n=354 | **0.046^†^** |
| NEC (>IIa)/FIP (%) | 5 (11.9) | 17 (4.5) | 0.057**^‡^** |

Total cohort: n = 420; missing data are indicated where applicable. DoxG2 (>75 - 250 mg/kg), ConG+DoxG1+3 = all other patients in our cohort. Non-normally distributed data are shown as median and interquartile range. Statistical tests used: ^†^Mann-Whitney U-test, ^‡^Fisher’s exact test. p < 0.05 was considered statistically significant and is shown in bold. BPD = bronchopulmonary dysplasia as defined by Walsh MC, Wilson-Costello D, Zadell A, et al., J Perinatol. 2003; FIP = focal intestinal perforation; IVH = intraventricular hemorrhage; NEC = necrotizing enterocolitis. ^§^Although the data were not normally distributed, mean ± SD (min-max) was reported since both median and interquartile range were zero.

**TABLE S3**. Number of neurodevelopment assessment tests applied in each group

| **Neurodevelopment assessment test** | **Control group n=178** | **Doxapram group n=111** | **DoxG1, n=42** | **DoxG2, n=32** | **DoxG3, n=37** | **P-value** |
| --- | --- | --- | --- | --- | --- | --- |
| Bayley II (%) | 87 (48.9) | 54 (48.7) | 21 (50.0) | 14 (43.8) | 19 (51.4) | ns |
| Bayley III (%) | 62 (34.8) | 33 (29.7) | 12 (28.6) | 9 (28.1) | 12 (32.4) | ns |
| Griffith (%) | 29 (16.3) | 24 (21.6) | 9 (21.4) | 9 (28.1) | 6 (16.2) | ns |

Statistical tests used: Fisher’s exact test. p < 0.05 was considered statistically significant.

**TABLE S4.** Psychomotor developmental outcomes in ConG and DoxG groups after 24 months corrected age, including dose subgroup analysis

| **Parameter** | **Doxapram group (n=140)** | **Control group (n=280)** | **DoxG1 (n=53)** | **DoxG2 (n=42)** | **DoxG3 (n=45)** | **P-value (DoxG vs. ConG)** | **P-value (ConG vs. DoxG1 - 3)** |
| --- | --- | --- | --- | --- | --- | --- | --- |
| PDI | 107.6 ± 12.5, n=17 | 96.9 ± 9.6, n=46 | 108.1 ± 7.9, n=7 | 101.0 ± 6.2, n=4 | 111.5 ± 18.6, n=6 | **0.005** | ConG vs. DoxG1: 0.046  ConG vs. DoxG2: 0.8  ConG vs. DoxG3: 0.03 |

Psychomotor developmental outcomes are presented for the Control group (ConG), the overall Doxapram group (DoxG), and for the Doxapram subgroups stratified by cumulative intravenous equivalent dose: DoxG1 (≤75 mg/kg), DoxG2 (>75 - 250 mg/kg), DoxG3 (>250 mg/kg). Missing data are indicated where applicable. The Psychomotor Development Index (PDI) of the Bayley Scales of Infant Development III was normally distributed and analyzed using an independent samples t-test for comparisons between ConG and DoxG, and one-way ANOVA for comparisons between ConG and the three dose subgroups. Tukey’s posthoc test was applied.

**TABLE S5.** Clinical characteristics of Doxapram-treated preterm infants with MDI/GDQ ≥ 70 compared with those with severe neurodevelopmental impairment (MDI/GDQ < 70; >2 SD below the mean)

| **Parameter** | **Doxapram group**  **MDI/GDQ ≥ 70 (n=98)** | **Doxapram group**  **MDI/GDQ < 70 (n=13)** | **P-value** |
| --- | --- | --- | --- |
| Gestational age (weeks) | 25.8 (24.6 - 27.3) | 25.7 (23.9 - 27.0) | 0.33^†^ |
| Birth weight (kg) | 0.81 (0.67 - 0.97) | 0.65 (0.58 - 0.73) | **0.0075**^†^ |
| Small for gestational age (birth weight <10^th^ percentile) | 13 (13.3) | 4 (30.8) | 0.11^‡^ |
| Weight at discharge <10^th^ percentile (%) | 40 (40.8) | 11 (84.6) | **0.006^‡^** |
| Head circumference at birth ≤ 3^rd^ percentile | 3 (3.1) | 1 (7.7) | 0.4^‡^ |
| Head circumference at discharge ≤ 3^rd^ percentile | 32 (32.7) | 7 (53.9) | 0.21^‡^ |
| Sedation required (Morphin and/or Fentanyl and/or Midazolam) > 48h (%) | 19 (19.4) | 1 (7.7) | 0.46^‡^ |
| IVH ≤II° or cerebellar hemorrhage (%) | 18 (18.4) | 7 (53.9) | **0.009^‡^** |
| Blood stream infection (%) | 2 (2.1) | 3 (23.1) | **0.01^‡^** |
| NEC (>IIa)/FIP (%) | 4 (4.1) | 3 (23.1) | **0.03^‡^** |
| Invasive mechanical ventilation: S-IMV+HFO [h] | 136 (8 - 292) | 307 (150 - 508) | **0.03^†^** |
| Moderate or severe BPD (%) | 43 (43.9) | 7 (53.9) | 0.56^‡^ |
| Dexamethasone treatment (%) | 26 (26.5) | 5 (38.5) | 0.51^‡^ |

Total cohort: n = 111; values are presented as median (interquartile range; 25. - 75. percentile) due to non-normally distribution or as n (%). Statistical tests used: ^†^Mann-Whitney U-test, ^‡^Fisher’s exact test. p < 0.05 was considered statistically significant and is shown in bold. BPD = bronchopulmonary dysplasia as defined by Walsh MC, Wilson-Costello D, Zadell A, et al., J Perinatol. 2003; FIP = focal intestinal perforation; HFO = high frequency oscillation; IVH = intraventricular hemorrhage; MDI = Mental Development Index (from the Bayley Scales of Infant Development II and III); GDQ = Griffith’s Developmental Quotient; S-IMV = synchronized intermittent mandatory ventilation.

**TABLE S6**. Clinical characteristics of the overall study cohort, comparing preterm infants with MDI/GDQ ≥ 70 and those with severe neurodevelopmental impairment (MDI/GDQ < 70; >2 SD below the mean), irrespective of Doxapram exposure

| **Parameter** | **Overall cohort**  **MDI/GDQ ≥ 70 (n=274)** | **Overall cohort**  **MDI/GDQ < 70 (n=15)** | **P-value** |
| --- | --- | --- | --- |
| Gestational age (weeks) | 28.1 (26.0 - 30.0) | 25.9 (24.0 - 27.0) | **0.0006^†^** |
| Birth weight (kg) | 1.0 (0.8 - 1.3) | 0.7 (0.6 - 0.8) | **<0.0001**^†^ |
| Small for gestational age (birth weight <10^th^ percentile) | 44 (16.1) | 4 (26.7) | 0.29^‡^ |
| Weight at discharge <10^th^ percentile (%) | 40 (14.6) | 12 (80.0) | **0.006^‡^** |
| Head circumference at birth ≤ 3^rd^ percentile | 9 (3.3) | 1 (6.7) | 0.42^‡^ |
| Head circumference at discharge ≤ 3^rd^ percentile | 65 (23.7) | 7 (46.7) | 0.06^‡^ |
| Sedation required (Morphin and/or Fentanyl and/or Midazolam) > 48h (%) | 27 (9.9) | 1 (6.7) | >0.99^‡^ |
| IVH ≤II° or cerebellar hemorrhage (%) | 47 (17.2) | 8 (53.3) | **0.0023^‡^** |
| Blood stream infection (%) | 11 (4.0) | 3 (20.0) | **0.03^‡^** |
| NEC (>IIa)/FIP (%) | 11 (4.0) | 3 (20.0) | **0.03^‡^** |
| Invasive mechanical ventilation: S-IMV+HFO (h) | 8 (0 - 114) | 222 (121 - 500) | **<0.0001^†^** |
| Moderate or severe BPD (%) | 49 (17.9) | 7 (46.7) | **0.005^‡^** |
| Dexamethasone treatment (%) | 34 (12.4) | 6 (40.0) | **0.009^‡^** |

Total cohort: n = 289; values are presented as median (interquartile range; 25. - 75. percentile) due to non-normally distribution or as n (%). Statistical tests used: ^†^Mann-Whitney U-test, ^‡^Fisher’s exact test. p < 0.05 was considered statistically significant and is shown in bold. BPD = bronchopulmonary dysplasia as defined by Walsh MC, Wilson-Costello D, Zadell A, et al., J Perinatol. 2003; FIP = focal intestinal perforation; HFO = high frequency oscillation; IVH = intraventricular hemorrhage; MDI = Mental Development Index (from the Bayley Scales of Infant Development II and III); GDQ = Griffith’s Developmental Quotient; S-IMV = synchronized intermittent mandatory ventilation.
